# The impact of improved road networks on marketing of vegetables and households' income in Dedo district, Oromia regional state, Ethiopia

**DOI:** 10.1016/j.heliyon.2021.e08173

**Published:** 2021-10-13

**Authors:** Abdo Wudad, Sultan Naser, Latamo Lameso

**Affiliations:** a Department of Geography and Environmental Studies, Kebri Dehar University, Ethiopia; b Department of Civic and Ethical Education, Mizan Tepi University, Ethiopia; c Department of Natural Resource Management, Kebri Dehar University, Ethiopia

**Keywords:** Livelihood, Income, Market access, Road infrastructure, Vegetable production

## Abstract

Rural infrastructures are important factors which are involved in agricultural development in Ethiopia. Among them, rural road facilities play a very significant role in the improvement of agricultural production and household income. This is because a good rural road network hurries efficient delivery of agricultural farm input and creates an opportunity to supply product to market. Currently, poor road conditions are hindering supply of product to market, which in turn affects households' annual income in most rural areas of Ethiopia. Therefore, this study aimed to asses the impact of improved road networks on marketing of vegetables and households' income in Dedo district in Ethiopia. For the study, two kebele were selected and data were collected from randomly selected 176 households from two kebele in the district. In addition to this, key informant interviews and focus group discussions were also conducted. Data were analyzed by multiple response tests and multiple linear regression models on statistical packages of the social sciences (SPSS). Study found that, from the total annual income of households; 58.5% of income was earned from vegetable production and it takes a lion share of households’ annual income in the study area. Regression results revealed that independent variables in the study had an insignificant influence on rural household annual income (p < 0.05). The multiple correlation coefficient measure (R = 0.845) also indicates that the relationship between rural household annual income and independent (set of explanatory) variables was strongly correlated. Findings also expose that high transportation costs incurring, spoilage of the product, deprived extension, service and market information, and reduction of household income are among the major impacts of road infrastructure in the district. Therefore, study suggested that rural households must have gained road access and federal and local road authorities should give attention to rural area road infrastructural development.

## 1 Introduction

Rural infrastructures which comprise rural roads, markets, health and educational facilities are among the basic to quality of life in rural areas. These infrastructures play a very significant role in accelerating agricultural production and product marketing. This is because a good road network hurries efficient delivery of agricultural farm input, reduces transportation costs, and improves agricultural production and distribution (Inoni and Omotor, 2009). Road infrastructure is among agricultural infrastructure which accelerates agricultural production in which rural roads connectivity is one of the key components for rural development. It promotes access to economic and social services, generating increased agricultural income and productive employment, as well as opening up new areas to economic focus (Lokesha and Mahesha, 2016; Tunde and Adeniyi, 2012). Road infrastructure plays a vital role in improving the livelihoods of rural people in sub-Saharan Africa (Gina, 2013). Likewise, rural transport is important for the evacuation and marketing of farm products and the delivery of farm inputs and extension services, as it also expands production and raises incomes of rural households (Stephen, 2015).

Improving rural roads is thus a critical, and priority link to farmers to towns to facilitate market service of smallholders in which undoubtedly transport is important for marketing agricultural products (Adugna, 2009). The level of public infrastructure, especially roads, seems low in the rural areas where the majority of poor people live in the world. Poor road accessibility and inadequate roads put high costs on transportation, hence reducing the ability to access high quality inputs and limiting the use of local markets to the sales of their products (Adugna, 2009; Kishor and Basanta, 2021). It also hinders the purchase of consumer goods and opportunities for off-farm employment (Inoni and Omotor, 2009). Therefore, to create more opportunities to participate in the market economy and thereby raise them out of poverty; improvement of rural roads seems to be a clear means for the many rural people (Syviengxay, 2008).

In Ethiopia, road transport is the dominant mode and accounts for 90 to 95 percent of motorized interurban freight and passenger movements (Ibrahim, 2011). However, provision of infrastructure has remained one of the formidable challenges for rural areas of Ethiopia in its endeavor towards socioeconomic development and poverty reduction because of limited road networks (ERA, 2008). The low road density and seasonal state of the road raises constraints on rural producers (Sileshi and Tebarek, 2017; Jemal and Genet, 2019). Many people live, and produce far away from major roads, markets and to other socio-economic service centers. Consequently, smallholder agricultural producers face high transportation costs because of poor road conditions that raise prices of inputs, and impair further access to market, which leads to low productivity, education and health, which in turn hinder economic growth (Ibrahim, 2011). Poor conditions as well as lack of basic infrastructure such as roads and access to transport services make it difficult for poor people to access markets and its services (Starkey and Hine, 2014).

The study area is widely known for its vegetable production, which is the basis of livelihood and income sources of the local communities. This is because vegetables are considered to be a very important crop both from the point of food and economic value in providing a cheap source of nutrients and vitamins (Kishor and Basanta, 2021). The district is characterized by different types of vegetable production such as potato, tomato, carrot, onion, avocado, cabbage, which are used for many purposes including for food, for commerce, and also as source of income. Although the district is one of the areas widely known for agricultural production, which is the basis of livelihood and income sources for the communities, it is the most affected by poor road infrastructure problems. This poor road condition is hindering supply of vegetable products to market, which in turn affect household's livelihoods and income (DDFED, 2016). Contrary to this, providing farmers with good road access will boost agricultural production and improve rural household income (Wondemu, 2015).

Infrastructural limitations have imposed severe constraints on the distribution of fruits and vegetables to market (Bisht, 2013). Poor road networks accompanied by lack of market information have contributed to significant losses of horticultural products (Njaya, 2014). According to the study of Ji et al. (2018) density of road (low grade) has positive externality of the vegetable production in neighboring provinces. Road connectivity of the land to the road head is also a big problem (Bisht, 2013; Njaya, 2014) and it causes huge amount of money is involved in transporting the goods locally (Bisht, 2013). The major vegetable production and marketing system in Ethiopia is characterized by many constraints (Mengesha, 2015). It is facing different problems such as high postharvest losses; lack of genuine, timely market information, poor marketing infrastructures (Mengesha, 2015; Bezabih et al., 2015; Sileshi and Tebarek, 2017; Jemal and Genet, 2019). Therefore, market performance and the challenges of the market that households face affects the decision and extent of farmer participation on vegetable production, type of vegetable crops that they would like to grow, and the size of farmland they would like to allocate for vegetable production (Meron, 2015). This indicates the extent to which road infrastructure directly as well as indirectly affects production, supply system-to-market and income of the communities at the country level.

Investigation and identification of these road infrastructure-related problems at community level would identify gaps; give direction, and guide policy makers and development practitioners to make informed decisions. It would help for further improvement of road facilities for input delivery, supplying vegetable products to market and enhancing income of rural households through rural to-urban linkage. Moreover, the studies conducted on impact roads on the whole agricultural production failed to specify a short seasonal vegetable production system and determine the impact of road access on income of rural households. In line with this, the following two major hypotheses were drawn for this study: 1; Road infrastructure factors have positive and significant influence on vegetable production and marketing and 2; Road infrastructure factors also have positive and significant influence on rural household income in Dedo district.

## 2 Materials and methods

### 2.1 Description of study area

**Figure 1:**
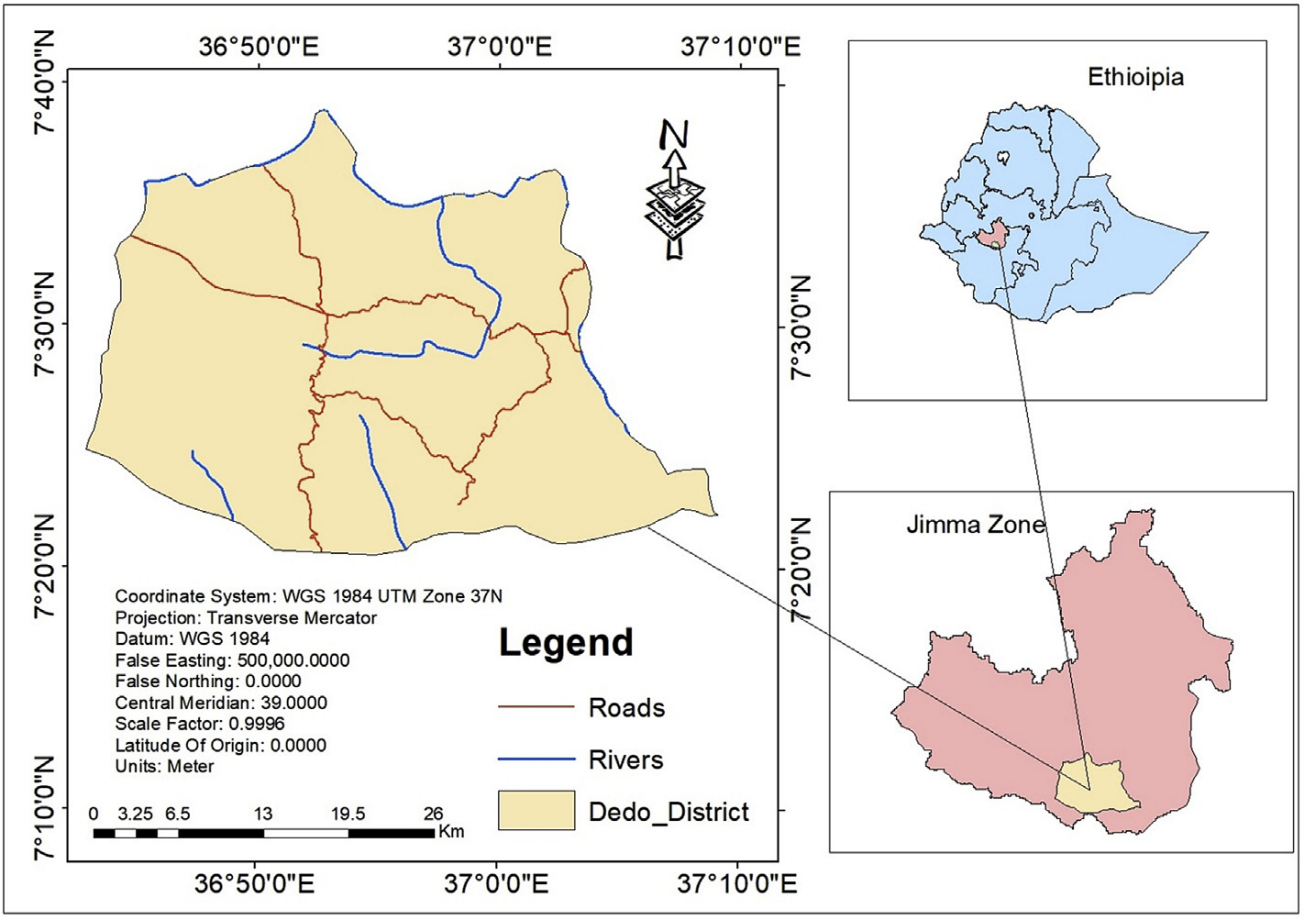
Map of the study area.

Dedo district is one of the 21 districts found in the Jimma zone of Oromia Regional National State. Geographically it is located between 7°5′-7°45′ N and 36°39′-37°15′E. It is bordered by Kersa district in the north; Mancho district in the east; South nations and nationalities regional state in the south, and Seka Chokorsa district in the west. The total surface area of the district is 797.8 square kilometers (Figure 1). The relief of the district is found within the south-western highlands of Ethiopia; its altitude ranges from 880 to 3046 m above sea level. It has three agro-ecological zones namely; highland (Dega) (32.6%), Midland (Woinadega) (49.2%), and lowland (Qolla) (18.2%). Recently the district was divided into 36 *kebeles;* from these 33 *kebeles* are peasant associations and the rest 3 are urban centres. Sheki is the capital town of the district. Because of geographical location, the district is near to the largest market center of Jimma town which has a great advantage for accessing the local products to the market as well as increasing sources of income and creates ideal conditions for provision of the demanded commodities to the local communities.

The climate of the district is tropical in nature and so experiences high incoming solar isolation due to high angle of the solar rays with overhead sun twice a year. However, this tropical nature of its climate is rather modified by altitude (Abdo, 2018) and central parts of the district have a cool agro-climate with the mean annual temperature ranges between 15^0^c -18^0^c. While the vast part of the district is classified as sub-tropical with mean annual temperature ranges between 18^0^c -29^0^c. The minimum temperature of the district is 11.27^0^c and the maximum temperature is 28.99^0^c. The rainfall of the district is weakly bimodal with spring having a small rainy season during the months of March and April, while summer a long rainy season during the months of June, July, August and September. Annual rainfall varies between 1,300mm and 1,700mm in major areas of the district (DDFEDO, 2019).

The district is rich particularly for farming practice because it has ideal agro-climatic conditions that are suitable for production of cereals. The livelihood of rural people is dependent on crops (DDFEDO, 2019). In addition to this, the district is widely known for vegetable production, which is the basis of livelihood for the communities as well as the economy of the district. The district has the annual total capacity of more than 380,360 quintals of vegetable production such as potato, tomato, carrot, onion, cabbage, which is used for many purposes including for food, for commerce, and also as source of income. This makes the vital role of vegetable production immense and it contributes more than 43,000 Ethiopian Birr tax in the district annually (DDFEDO, 2019).

The road facilities of the district are so poor and have adverse effects on supply of these resources of vegetable production which in turn affect household livelihoods and income and also lead to high costs, different losses of the traders and income of household farmers. Nevertheless, the district has a large potential for passengers in which at an average of per 10 min one vehicle is transported and 30–34 cars per day are used with 1200–1500 average number of passengers transported only to one route from Dedo to Jimma. However, due to poor condition of road infrastructure it results in high transportation cost, which might impact rural household income (DDRTO, 2020).

### 2.2 Research design

In order to answer the research questions, the study employed a mixed research method due to the fact that it helps to triangulate qualitative and quantitative data in relation to the impacts of road infrastructure on vegetable product marketing and households' income. The mixed method was mainly used in this study to have better insights and understanding about the impacts of road networks on marketing of vegetables and households' income and to broaden the extent of analysis from the findings of the study. Regarding this, Akimowicz et al. (2018) suggest that agricultural researchers and economists would benefit from using mixed methods to enhance the breadth and depth of their analysis. Therefore, a qualitative method was used to provide detailed information which can better explain relationships observed from quantitative methods. Accordingly, all necessary data were gathered through semi structured questionnaires, key informant interviews and review of different documents which are related to road infrastructure, vegetable market and rural household income. The collected data were analysed by involving both qualitative and quantitative methods of data analysis.

### 2.3 Sampling techniques and sample size determination

Both purposive and random sampling techniques were employed for the study. Purposive sampling technique was used to select the study district and Kebele (smallest administrative unit) and random sampling techniques were used to select sample respondents for the interview. Following this, Dedo district was purposely selected from 23 districts of Jimma zone, due to the reason of widely existence of vegetable production and suffering of the communities from poor road infrastructure problem as consultation with of Jimma zone agricultural office and development agents. Then, two potential vegetable producers’ *kebeles*, namely, Geshe, and Sito, were selected purposively from 33 kebeles in the district because they are predominantly engaged in vegetable production (DDFEDO, 2019).

The two sample Kebeles had a total of 1354 household heads from which the household population of Geshe, and Sito were 623 and 731, respectively. To keep the representativeness of the sample in the population, the study used the formula given by Kothari (2004) for sample size determination (equation 1).

(1) $$\text{n}=\frac{z^{2}.P.q.N}{e^{2}(N-1)+z^{2}P.q}$$

Where, n = desired sample size, z = standard normal deviate at the required (95%) confidence limit (1.96), p = is 0.05 (proportion of the target population to be included in the sample, q = 1-p (1-0.05 = 0.95), e = level of statistical accuracy set at 0.03 and N = total number of population.

$$\text{n}=\frac{1,96^{2}x0.05x0.95x1354}{0.03^{2}\left(1354-1\right)+1,96^{2}\;0.05x0.95}=176$$

Having the selected sample size, 81 and 95 respondents were randomly selected from Geshe and Sito *Kebeles* respectively by using proportional probability.

### 2.4 Method of data collection

Numbers of data collection methods were used and all necessary data were gathered through respondent interview, key informant interview, focus group discussion and observation. Data from household respondents were collected through using a semi-structured questionnaire which included both open-ended and close-ended questions as modified by (Catherine, 2002). The household questionaries’ was basically conducted to collect data from the selected sample households regarding vegetable production and impact of road infrastructure on their income. The study also incorporated six different focus group discussions at district level to gather additional data to substantiate the data collected through questionnaires. The focus group discussions were conducted with a group of farmers that constitutes 6–8 groups of elderly people with the age of more than fifty and young women and man with the age between twenty five and thirty five. In addition, key informant interviews were held with different experts from rural road authority of the district, agricultural office of the district, market development office of the district, trade office of the district and development agents (DA) in order to get rich information about the impact of improved road networks on vegetable marketing and rural household income for the sake of supporting the quantitative data.

### 2.5 Methods of data analysis

The data generated through questionnaires, key informant interviews and focus group discussions were analyzed and interpreted qualitatively and quantitatively. Qualitative data were analyzed through descriptive statistics, whereas quantitative data were first recorded and organized in the Statistical Package of the Social Sciences (SPSS Version 23) and analyzed using multiple response tests and multiple linear regression models. Multiple linear regressions were used to analyze the impact of independent variables whose values were known to predict the single dependent variable. As explained by Mohamed (2015) it was used because it allows determining the overall fit of the model and the relative contribution of each of the predictors to the total variance explained.

### 2.6 Multiple linear regression model specification

Linear regression analysis is a statistical method used to estimate the relationship between a single dependent variable and one or more independent variables (Mohamed, 2015). A multiple linear regression method was applied to determine significant factors from potential explanatory variables. Independent variables include personal characteristics, socio-economic factors and institutional factors that may influence the dependent variable. In this study, dependent variable was rural household income and independent variables were road infrastructural factors that were identified as negatively affecting income of rural households (Jemal and Genet, 2019; Ina et al., 2019) were considered. The general form of a multiple linear regression model is shown using the following formula.

(2) Y = β_o_ + β_1_ X_1_ + β_2_ X_2_ + β_3_X_3_ … + β_k_ X_k_ + εi

Where, Y = Dependent variable, X_1_ to X_9_ = Independent variables, β_1_ to β_9_ = Coefficients of independent variable, Β_0_ = intercept and εt = error term. Depending on multiple linear regression equations which were employed by (Gary, 2003; Alvin and Bruce, 2008; Erik and Sarstedt, 2014) the relationship between the dependent and independent variables of our interest were represented as follows.

RHI = f (DHM, DHNMR, RST, AMI, FCR, TRA, MMT, TTHM and CRA). Where,

Y = RHI (Rural Household Income) continues variable.

X_1_ = DHM (Distance from Home to Market (km)) Categorical variable.

X_2_ = DHNMR (Distance from Home to Nearest Main Road (km)) Categorical variable.

X_3_ = RST (Road Surface Type) Categorical variable.

X_4_ = AMI (Access to Market Information) dummy variable.

X_5_ = FCR (Functionality Classification of Road) Categorical variable.

X_6_ = TRA (Type of Road Accessibility) Categorical variable.

X_7_ = MMT (Major Means of Transportation) Categorical variable.

X_8_ = TTHM (Travel Time from Home Market (hrs)) Categorical variable.

X_9_ = CRA (Condition of Road Access) Categorical variable.

By relying on the functional form of the relationship between variables of interest above, multiple regression model was developed as follows (equation 3).

(3) Yt = β_0_+ β_1_ DHM + β_2_ DHNMR + β_3_ RST + β_4_ AMI +β_5_ FCR + β_6_ TRA + β_7_ MMT + β_8_ TTHM + β_9_ CRA +εt

#### 2.6.1 Assumption test (model output diagnosis)

The major assumptions in the multiple regressions such as normality, linearity, multicollinearity and autocorrelation were checked for model diagnosis. Accordingly, the relationship between the independent variables and the dependent variable is linear, and the P–P plot shows that this assumption had been met (appendix 2). The values of the residuals are normally distributed and the histogram for the model suggests that the assumption of a normal distribution of the residuals has been met (appendix 3). Furthermore, the plot of standardized residuals vs standardized predicted values showed that the variance of the residuals is constant or roughly similar, which indicates that the assumption of homoscedasticity has been met (appendix 4). The Durbin-Watson statistic also showed that the values of the residuals were independent so that this assumption had been met, as the obtained value was close to 2 (Durbin-Watson = 1.875) (appendix 1). The values of variance inflation factor (VIF) were less than 5. Based on the VIF result, the data had no serious problem of multicollinearity or there is no multicollinearity in the data, which implies that the predictors (explanatory/independent variables) are not too highly correlated.

Multiple correlations were used to check the autocorrelation of the variables and found that the relationship between rural household annual income (continuous variable) and independent (set of explanatory) variables was strongly correlated. The value of the coefficient of determination (R^2^) implies that about 71% of the total variation in household annual income was explained by the independent variables in the model. The adjusted R squares were about 70% this indicates the strongest predictive power of the independent variables over the dependent variables of the study. The Durbin-Watson test implies values approximately around 2 (1.9) indicate no autocorrelation of the linear regression model (appendix 1). In addition, ANOVA was used to test whether, the multiple linear regression models fitted to show the influence of a set of explanatory variables on the single dependent variable. Accordingly, the ANOVA result (F = 46.097; P < 0.05) showed that there is a good model fitting, thus, entails that the explanatory variables included in the model jointly influenced total household income (appendix 1).

## 3 Results and discussions

### 3.1 Rural household production system and income sources in the study area

The study found that agricultural activities are the main means of livelihoods of many rural households and major sources of income in the study area. According to respondents, the major agricultural activities and income sources of households are crop production and livestock rearing. As respondents pointed out, these two agricultural activities and off-farm income sources were the sources of income for rural households in the study area. Quantitative data obtained from sample households from study areas shows that mean annual income was 52,908.11 Ethiopian Birr (ETB). Most rural household income was generated from agricultural or farm activities (Table 1). The farming communities in the study area primarily depend on agricultural activities, especially on vegetable production, which plays an important role in their livelihood as well as total household income. Response from respondents and key informants (KIs) showed that the crops produced in the study area were cereals, pulses, fruits, vegetables and cash crops. According to them, vegetable production is the major means of a rural household's livelihood and it takes line share of annual income earned from agricultural activities. The major vegetables produced in the area are, potato (*Solanum tuberosum*), tomato (*Solanum lycopersicum*), cabbage (*B. oleracea* var. *capitata*), carrot (*Daucus carota*), onion (*Allium cepa*), mustard/kale (*Brassica carinata*), beetroot (*Beta vulgaris*), Sweet potato (*Ipomoea batatas*) and chili (*C. chinense*). This is because various types of vegetable crops are grown in Ethiopia under rain-fed and/or irrigation systems (Alemayehu et al., 2010).

**Table 1:** Source of income and amount of each source in Ethiopian birr.

Rural household income sources	N		Minimum	Maximum	Mean
	Valid	Missing			
Income from crop production	176	0	16,450	39,200	30,986.28
Income from livestock	146	30	1,000	8,500	3,348.63
Income from off farm and nonfarm	176	0	17,000	23,500	19,138.59
Total income	176	0	36,450	65,900	52,908.11

According to respondents, vegetable production is integrated ​into a mixed ​farming system where different types of crops are produced on the same plot of land or in sequence with other crops in rotation. The respondents also argued that, some vegetables are grown either solely or intercropped with other vegetables or cereals, depending on availability of land and crop suitability for intercropping. ​Respondents revealed that they ​primarily, utilizing their income on purchasing food, clothes, education fees and other basic needs. According to ​them, the ​majority of vegetables are also used for household consumption. Similarly, a study ​from Kishor and Basanta (2021) ​found that ​farmers are utilizing their income from vegetable production for food purchasing, children's’ education and clothes and other daily required goods.

Respondents pointed out that livestock production is also an important productive asset next to vegetable production and an important source of income for smallholder households. The main kinds of livestock available in the study area are cattle such as cows, oxen, sheep, goats, donkeys, horses and mules. These domestic animals have a crucial role in the livelihoods and source of income of the community through providing power for traction and transportation of food, fertilizer and cash earning from renting and selling both the products and live animals. Similar to this finding, Herrero et al. (2013) found that livestock play a significant role in rural livelihoods and the economies of a shift to more non-farm income in comparison with farms.

Respondents revealed that off-farm and nonfarm activities were also other sources of household income. An off-farm activity refers to agricultural activities which take place outside the person's own farm. The activities include local daily wage labor at the village level or the neighboring areas in return for cash payment at another person's farm. On the other hand, nonfarm activities or income sources refer to activities that take place outside the agricultural sector, which includes handicraft activities and petty trade. According to Yenesew et al. (2015) handicraft activities such as weaving, spinning, carpentry, house mudding, poet making, remittance and petty trade such as grain trade, fruits and vegetables trade and selling of local drinks are the major nonfarm activities.

### 3.2 The nature of roads access in the study area

The physical condition of the road surface of the study area is one of the road infrastructural factors that are assumed to influence or affect rural household income. Therefore, the main road infrastructural factors such as types of road, physical conditions of road, their functional classification and distance from home to market were identified and described. The types of road which were described by participants in the key informant interview from the district road and transport office indicate that only 4.5% is concrete road; 39.8% is gravel road and more than 55% is earth road (Table 3). The perception of respondents’ on the physical condition of roads indicated that only 6.8% of roads are good, whereas 21.6 % and 71.6 % of road physical condition are fair and poor, respectively (Table 2).

**Table 3:** The nature of roads access in the study area.

Nature of roads access	Category	%
Types of road	Concrete road	4.5
	Gravel road	39.8
	Earth road	55.7
Functional road classification	Trunk	7.4
	Link	7.4
	Main access	35.2
	Collector	10.2
	Feeder road	35.8
	Paved road	4.0

**Table 2:** Nature of road and related factors.

Road related factors	Perception of respondents	n = 176	
		Frequency	%
Physical condition of roads	Good	12	6.8
	Fair	38	21.6
	Poor	126	71.6
Distance from home to market	Less than 2	10	5.7
	2–3km	10	5.7
	4–5km	116	65.9
	6–7km	38	21.6
	8 or more km	2	1.1
Access to market information	Yes	19	19.9
	No	81	80.1

Distance from home to market is also another road-related factor which has an influence on household income. Regarding to this more than 65.9% of the respondents revealed that the distance from their home to the next main road was about 4–5km and 21.6% of respondents exposed that the distance from the nearest main road to market is 6–7km and the rest of respondents replied that the estimated distance of their home from the nearest main road is 5.7%, 5.7% and 1.1% was <2, 2–3, and ≥8km, respectively (Table 2). Road functional classifications such as main access road, collector, feeder, trunk, link and paved road are also road infrastructural factors that are believed to influence rural household income.

The ​district ​road and transport office ​ ​experts in ​the key informant interviews indicated ​that 35.8% of roads in the study area are feeder roads and 35.2% are ​main access roads, while 10.2%, 7.4%, 7.4%, and 4% are collector, trunk, link, and paved roads, respectively (Table 3).

The nature of road infrastructure was assessed in different ways using several road infrastructural factors. ​The results of the study revealed that suitable roads and road infrastructure are poorly accessed in the study area (Table 3). In addition to this, based on observed data, there is very limited access to ​the ​trunk road and ​the ​majority of respondents use feeder ​roads ​and main access. ​Participants in focus group discussion and key informants from ​district agricultural office, trade and industry office and office of market development of the district exposed ​that these road-related problems become bottlenecks for vegetable production and marketing. ​They revealed that it’s ​on decreasing labor ​force mobility and thereby ​declining ​households' job opportunities and ​causing a ​rise ​in the ​cost of transport. ​Respondents revealed that, lack of improvement of transport services incurs additional the transport cost of goods, which results in decrease in farm gate prices of agricultural products and has a negative influence on rural household income. ​According to respondents, this is because of increasing the prices of agricultural inputs and additional costs incurred during supply of product to market.

Respondents also believed that good road transport could further reduce production costs by lowering prices of delivered inputs, while at the same time it increases net farm gate prices and increasing farm income. It also shows that all-weather road access not only increases income from farming activities but also makes prices more stable and enables the poor to improve risk management and reduce risk. Similarly, with respect to the current discussion Torbjø rn and Bharat, 2012 explained that the poor and remote communities get larger benefits from a new road in several ways which means, road construction and maintenance might give employment opportunities for the local people; while on the other hand improved transport at same time reduces the physical costs of access to resources and markets.

On the other hand, market information is also another factor that is assumed to determine or have an impact on vegetable product market and rural household income. In this case, the results of the study revealed that only 19.9% of the vegetable producers have vegetable production market information and more than 80% have no vegetable product market information (Table 2). Similarly, the response from participants in focus group discussions explained that due to poor road infrastructure and other inaccessibility, it is difficult to provide timely information about the markets for all residents of the district. This in turn has adverse effects on income from vegetable production in the study area.

### 3.3 Major means of transportation

**Figure 2:**
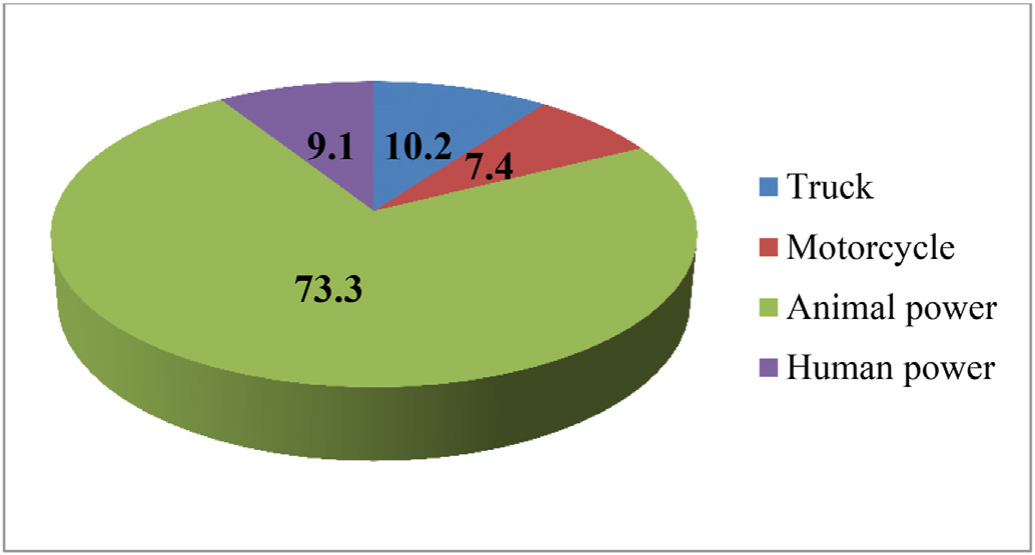
The major means of transport frequently used to supply production to market.

The major means of transportation used by the household to supply their product to market are the other road infrastructural factors which have an impact on rural household income. This study found that truck, motorcycle, human power and animal power are the means of transportation used by households to deliver agricultural inputs and to supply their products to market in the area. Regarding this, more than 73.3% of vegetable producers frequently use animal power and only 10.2% of them have the opportunity to use trucks (Figure 2).

Respondents directly relate this to the availability of suitable roads. According to them, when the quality of roads increases, they have more opportunity to use trucks for delivering input to farms and supplying products to market. From this result, it is clear to understand that means of transportation negatively affect the income of rural households. Similarly, a study by Usman et al. (2013) found that poor transportation has the negative effect of restricting expansion of agricultural production as farmers get very low farm-gate prices which are below the market price. Key informants also revealed that transporting the vegetable products into the roadsides and market by using animal power, requires longer time and is prone to spoilage vegetables, and it boosts the amount of wastage. This is directly affecting and causing the decline in annual income of rural households. Similar findings from Tunde and Adeniyi (2012) explain that major means of transportation because of bad condition of the roads limit the potential level of agricultural production.

### 3.4 The impact of road infrastructure on rural household income

The regression analysis on specific predictor variables were given in Table 4 below and show that rural household income is influenced by road associated factors such as distance from home to market, distance from home to nearest main road, road surface type, type of road accessibility, major means of transportation, travel time from home to market and condition of road because each of them significant at p-value less than 0.05, whereas functional road classification and access to market information showed insignificant influence on the rural household annual income as their p-values greater than 0.05 (Table 4).

**Table 4:** Result of multiple linear regression analysis.

Variables	Un-standardized Coefficients		Standardized Coefficients	T	Collinearity Statistics		Sig.
	B	Std. Error	Beta		Tolerance	VIF	
Constant	71856.786	2102.883		34.171	0.454	2.205	0.000
DHM	-3290.947	419.406	-0.483	-7.847	0.484	2.064	0.000
DHNMR	-2238.737	430.082	-0.310	-5.205	0.577	1.732	0.000
RST	-3951.895	496.235	-0.435	-7.964	0.930	1.075	0.000
AMI	-802.610	571.891	-0.060	-1.403	0.900	1.111	0.162
FCR	252.453	175.481	0.063	1.439	0.552	1.812	0.152
TRA	878.304	363.676	0.135	2.415	0.924	1.082	0.017
TTHM	-1191.974	351.130	-0.147	-3.395	0.893	1.120	0.001
MMT	1127.220	317.442	0.156	3.551	0.370	2.700	0.000
CRA	1392.558	599.016	0.158	2.325	0.454	2.205	0.021

The regression output results show that household total annual income has ​a ​positive and significant relationship with ​the ​condition of ​road accessibility. Being other variables constant, a high quality of road access conditions can increase the annual income of the households by a factor of 0.158 at p < 0.05 ​(Table 4). ​From this we understand that poor road access conditions lead to the less income earning of ​households ​and vice versa. Response of respondents also show good condition of quality road accessibility helps them to use truck ​and other transport options which ​help them to increase quality and quantity vegetable ​products ​as well as enable the households to ​easily ​find market access for their products. ​This is similar ​to the ​finding of ​Abur et al., 2015 ​who found that the coefficient of quality of rural roads had a significant influence on rural household income as it reduced home-to-market transportation costs. ​Similar findings from Hika (2017) also ​showed ​that better ​road accessibility could help farmers to get market information, which could encourage ​them ​to produce more for the future.

The regression results show that distance from home to market also has a significant influence on rural household annual income. Being other variables constant, a one km increment in the market distance decreases the annual income of the households by a factor of -0.483 at p < 0.05 (Table 4). As a result, being far from the market area leads to narrow opportunities for households to deliver agricultural inputs, the farm and supply their product to the market. So that it hinders household capacity to generate more income as well as to diversify their source of income. The findings of this study is in line with the study of Inoni, and Omotor (2009) and Aika et al. (2018) which reported that distance to market had significant impacts on rural households’ income. And also Yimer (2015) found that increase in distance from home to market distance influences rural household income through reducing the quantity of supplied agricultural products to the market.

The regression result for the variable of distance from home to nearest main road per km shows that household total annual income has a negative significant relationship. Being other variables constant, a one km increment of distance from home to nearest main road decreases the annual income of the households by a factor of-0.310 at p < 0.05 (Table 4). From this it is clear that, if there is an increased distance from the nearest main road, the less the income they generate, and vice versa. Respondents also revealed that when the main road far from home or production area declines, our income is earned through incurring additional cost post-production in the form of input delivery. Due to this, they are more forced to produce products that are needed for household consumption, which is scientifically called subsistence production. In addition to this, it incurs another cost through payment for labor and transport during supply. Study conducted by Ghirmai and Tesfayesus (2016) also revealed similar to this finding, that increase in distance to the nearest main road, which can be considered as an alternative measure for distance to the market, was found to have significant negative influence on income of house because of wage employment. Regarding this study conducted by Hika (2017) found that people nearest to the road have advantage to get market access and they are willing to produce more for commercial purposes, while those with poor market access because of long distance to market are forced to produce for domestic consumption.

Road surface type is also among the factors that influence marketing of vegetable production, which in turn affects rural household income. The regression output result for this variable shows that household total annual income has a negative significant relationship with type of road access. Being other variables constant, a categorical increment in one type of road surface decreases the annual income of the households by a factor of -0.435 at p < 0.05 (Table 4). Unlike the previous assumption that hypothesized the type of road surface might have a positive significant relationship with rural household income, this study found that to have a negative significant relationship with the dependent variable. This indicates that the income of households that have access to concrete roads was less than those that have access to gravel roads and earth road surface types. This may be due to the fact that the majority (73.3%) of the respondents in the study area uses animal power as the major means of transportation which didn't bother them on road quality. Contrary to this study done by Sileshi and Tebarek (2017) reported quality of road accessibility increases i.e from earth road to gravel road increases the productivity of smallholder farmers.

Types of road accessibility are also the other factors that influence rural household income. The regression result for this variable reveals that household total annual income has a positive significant relationship with types of road accessibility. Being other variables, constant, a one-increment in accessibility type of road increases the annual income of the households by a factor of 0.135 at p < 0.05 (Table 4). This indicates that poor type of road accessibility hinders income earned by the households and vice versa. Similar findings from Olusogo et al. (2018) confirm that an increase in road transport infrastructure leads to significant increases in agricultural output and agricultural sector development.

Traveling time from home to market is also the other major road infrastructural factor which affects rural household income. The regression results for this specific variable show that home-to-market travel time has a negative significant influence on household total annual income. Being other variables constant, 1 h increments in travel time decreased the annual income of the households by a factor of -0.147 at p < 0.05 (Table 4). High travel time from household home to market, leads to the less income earning of the household and the less travel time resulted in high income earned by the household. This is because of the long wastage of time while traveling to market which resulted in the short working hours of the households which in turn affected the productivity and income of the households. The similarity of Ghirmai and Tesfayesus, 2016 found that with every hour of increased distance to the market incurs additional costs, wage employment and non-agricultural wage employment, and also reduces farm working hours.

The major means of transport are also assumed to have an impact on rural household income from the vegetable product market. Based on this assumption, the regression results show that means of transport has a significant positive relationship with household total annual income. Being other variables, constant, change in means of transport from human and/or animal to motorcycle and/or truck, increase the annual income of the households by a factor of 0.156 at p < 0.05 (Table 4). This implies that the major means of transportation frequently used by households determine or affect their income because of the quantity of production and the time required to supply the products to market directly influence household income. Similar study from Tunde and Adeniyi (2012) and Usman et al. (2013) confirm that poor transportation has the negative effect on agricultural production and then reduces rural household income.

In nutshell, the above mentioned results from this study have an implication on the road networks improvement strategy and marketing of agricultural products. Findings of the study revealed that bad road networks in the study are increased transport cost, diminish efficient delivery of farm inputs and reduce agricultural production and supply. Strategies followed for development sectors rely on information and finding of the studies (Worku, 2010). Regarding to this, this study found that lack of qualified and improved road network has a negative influence on farmers' income as it incurs additional cost and resulting diminishing farm profit. Study also found required information which can be input for strategic plans at the district level for future improvement of the rural road network. In rural areas, improving rural road networks is among other strategies to stimulate agricultural sector development and farmers’ income (Fungo et al., 2017). In line with this, the finding of the study has advantage for rural road authority and agricultural office of the district as it can be used as a data source for strategies planning. This is because; planning and implementing based on finding of the study can boost production farm level and increase the rate and amount of supply of product to the market.

## 4 Conclusion

Agricultural production, specifically vegetable production, is the basis of livelihood and income sources of the local communities in Ethiopia. Even though vegetable production is one of the widely known agricultural productions which are major income sources for the communities, it is the most affected by poor infrastructural problems, especially those of roads. Poor conditions as well as a lack of basic infrastructure such as roads and access to transport services make it difficult for poor people to deliver inputs and access markets for supplying products. This is also the case for rural households in Dedo district. It is because vegetable production is among the main means of livelihood as well as source of income for them. The finding shows that road access conditions in the study area are generally poor, having significant effects on vegetable product markets and rural household income. The result of the study also reveals, road access condition, major means of transportation, type of road accessibility have significantly positive influence on rural household annual income, whereas the distance from home to market, category of road access, traveling time from home to market and home distance from the nearest main road have negative significant influence on rural household annual income. From identified impacts reduction on vegetable production because of reducing input delivery, incurring high transportation cost, causing spoilage of the product, poor market information access and extension service are among the major impacts of road infrastructure on vegetable product marketing and household income in the district.

## 5 Recommendations

The study recommended that rural road authorities and transportation offices have to provide a support through the improvement of road infrastructure projects and other agricultural production. Local administrative units and development agents should create possibilities for the farmers to have timely market information through different media. Both government and nongovernment organizations should give attention to supporting farmers through required input delivery with minimum purchasing and transportation costs. The government, cooperative organizations and private organizations should also give attention to the provision of adequate road infrastructural services and supply of agricultural inputs on time. An effective marketing system would facilitate production adoption; so other concerned bodies such as governmental extension services, market development office of the district, farmers’ cooperatives and non-governmental market organizations should support the development of efficient marketing systems to support vegetable production in the study areas. Finally, the government should assist the farmers to ensure their food security and to change livelihood as well as income of rural household farmers.

## Declarations

### Author contribution statement

Abdo Wudad Kemal and Sultan Naser Abagisa: Conceived and designed the experiments; Performed the experiments; Analyzed and interpreted the data; Wrote the paper.

Latamo Lameso Lelamo: Conceived and designed the experiments; Analyzed and interpreted the data; Wrote the paper.

### Funding statement

This research did not receive any specific grant from funding agencies in the public, commercial, or not-for-profit sectors.

### Data availability statement

Data included in article/supplementary material/referenced in article.

### Declaration of interests statement

The authors declare no conflict of interest.

### Additional information

No additional information is available for this paper.

## Appendix A Supplementary data

Appendixes Appendixes
