# Supplementary material for: The impact of improved road networks on marketing of vegetables and households' income in Dedo district, Oromia regional state, Ethiopia
Source: Heliyon. 2021 Oct 13;7(10):e08173. doi: 10.1016/j.heliyon.2021.e08173 (PMC8524753; doi:10.1016/j.heliyon.2021.e08173)

**Appendixes**

**Appendix 1: Model summary and ANOVA Regression Result**

**Model Summary**

| **Model** | **R** | **R Square** | **Adjusted R Square** | **Std. Error of the Estimate** | **Durbin-Watson** |
| --- | --- | --- | --- | --- | --- |
| 1 | 0.845^a^ | 0.714 | 0.699 | 2920.274 | 1.875 |

| **ANOVA regression result** | | | | | | |
| --- | --- | --- | --- | --- | --- | --- |
| Model | | Sum of Squares | Df | Mean Square | F | Sig. |
| 1 | Regression | 3538013608.144 | 9 | 393112623.127 | 46.097 | .000^b^ |
|  | Residual | 1415647690.805 | 166 | 8527998.137 |  |  |
|  | Total | 4953661298.949 | 175 |  |  |  |

**Appendix 2: Data linearity of multiple regression model analysis result**


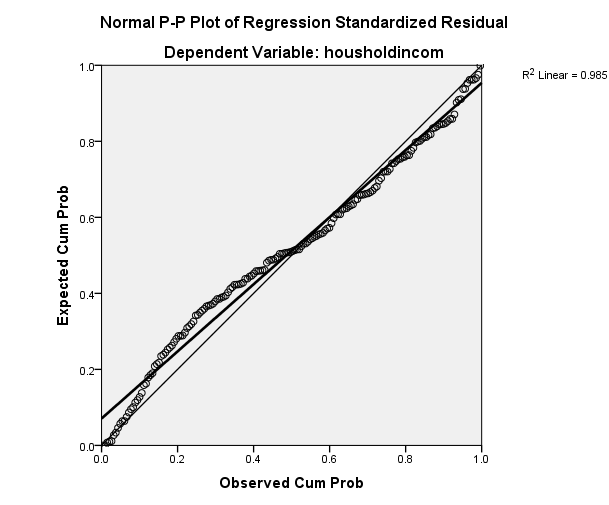


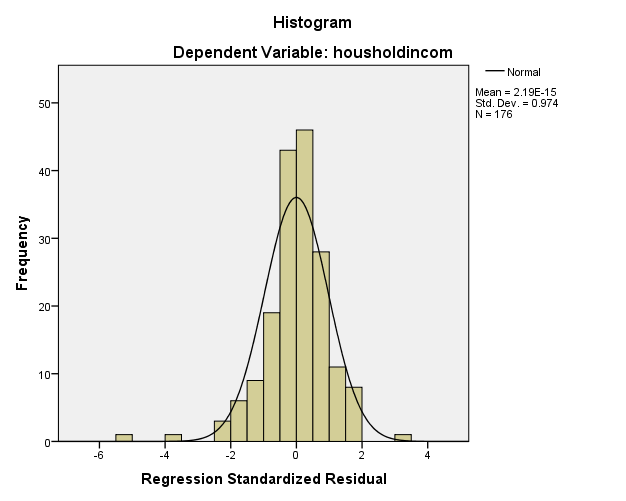
**Appendix 3: Data normality of multiple linear regression model analysis result**

**Appendix 4: Data homoscedasticity of multiple linear regression analysis**


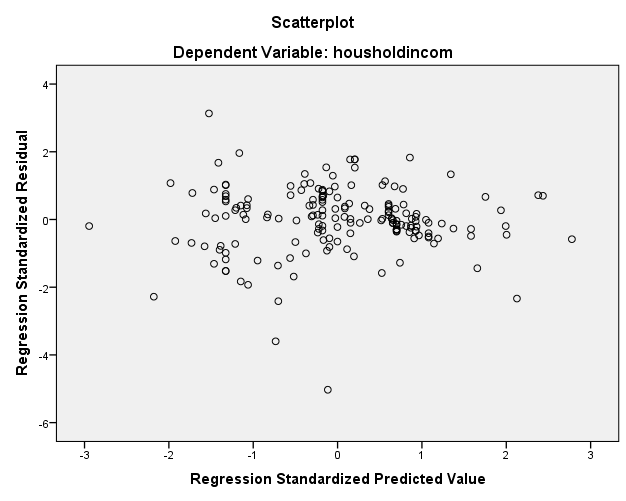

Supplement: Appendixes [file mmc1.docx]
